# Direct Oral Anticoagulants in Cirrhotic Patients: Current Evidence and Clinical Observations

**DOI:** 10.1155/2019/4383269

**Published:** 2019-01-08

**Authors:** Sherif Elhosseiny, Hassan Al Moussawi, Jean M. Chalhoub, James Lafferty, Liliane Deeb

**Affiliations:** ^1^The Department of Medicine at Staten Island University Hospital, Northwell Health, USA; ^2^The Department of Cardiology at Staten Island University Hospital, Northwell Health, USA; ^3^The Department of Gastroenterology at Staten Island University Hospital, Northwell Health, USA

## Abstract

The introduction of Direct Oral Anticoagulants (DOACs) to the pharmaceutical market provided patients and clinicians with novel convenient and safe options of anticoagulation. The use of this class of medications is currently limited to venous thromboembolic therapy and prophylaxis, in addition to stroke prophylaxis in patients with nonvalvular atrial fibrillation. Despite their altered hemostasis, patients with cirrhosis are thought to be in a procoagulant state and thus prone to thrombus formation. Patients with cirrhosis might benefit from the convenience of DOACs; however, the medical literature includes limited data on the efficacy and safety of DOACs in this special patient population. The aim of this review is to summarize the current evidence for anticoagulation options in patients with cirrhosis and their safety profile.

## 1. Background

Vitamin K antagonists (VKA) have been the standard of care for stroke prevention in atrial fibrillation, as well as venous thromboembolism (VTE) prevention and treatment. Given their narrow therapeutic index, close monitoring using the international normalization ratio (INR) is warranted.

The introduction of Direct Oral Anticoagulants (DOACs) in the past decade was a game-changer. DOACs are oral alternatives to Warfarin with a lower risk of bleeding that do not require recurrent blood draw for INR monitoring, and 1 has a quicker onset of action. Also, DOACs are beneficial in minimizing the confusion caused by the baseline elevated INR in patients with cirrhosis. The direct thrombin inhibitor Dabigatran, factor Xa inhibitors, Rivaroxaban, Apixaban, and Edoxaban were all approved for the treatment and prevention of VTE and the prevention of stroke in patients with nonvalvular atrial fibrillation. Several clinical trials aimed to evaluate the effectiveness and safety of DOACs compared to VKA in stroke prevention in atrial fibrillation and in the treatment of VTE. Compared to VKA, DOACs were shown to be noninferior in the treatment of VTE (reference). Dabigatran and Apixaban were shown to be superior to Warfarin in preventing strokes in patients with atrial fibrillation (reference). Compared to Warfarin, Rivaroxaban and Edoxaban were noninferior in preventing strokes in atrial fibrillation patients [1–6]. Other indications for DOACs include cancer associated thrombosis, secondary prevention of major cardiovascular disease in patients with acute coronary syndrome, prophylaxis of VTE in acutely ill hospitalized patients, and long-term management of ACS [7–10].

So far, most of the randomized clinical trials studying DOACs have excluded patients with remarkable liver disease and cirrhosis. The rationale behind cirrhosis being an exclusion criterion goes back to the complex hemostasis in patients with liver cirrhosis. As a result of hepatic dysfunction, patients have a “rebalanced hemostasis” leading to both procoagulant and anticoagulant effects [11]. Though patients with cirrhosis might have a prolonged INR and thrombocytopenia, studies showed an increased risk of VTE in this patient population [12]. Sogaard et al. found a 1.7-fold higher relative risk of VTE in cirrhotic patients compared to healthy controls. On the other hand, in cirrhotics, especially those with large varices or varices with red wale sign, variceal bleeding remains a substantial concern [13, 14]. Nowadays, given the lack of high-level evidence, common practice is to assess the individual risks and benefits of anticoagulation when indicated in this subset of patients.

Of all anticoagulants available, low molecular weight heparin (LMWH) and Warfarin with INR target between 2 and 3 were the most studied and used in patients with cirrhosis. The use of DOACs in patients with cirrhosis is still not a common practice given the limited data about their efficacy and safety in this special population [15]. The aim of this review article is to discuss the current evidence of potential indications and safety of DOACs use in patients with liver cirrhosis.

## 2. Hemostasis in Liver Disease

Several defects account for the altered hemostasis in patients with liver disease. A recent study concluded that cirrhotic patients have an overall procoagulant state but a decreased clot formation capacity and an unaltered resistance to clot lysis [16]. Most reported hemostatic abnormalities are related to the production of pro-, and anticoagulation factors, the associated thrombocytopenia and platelet dysfunction, and the increased fibrinolysis.

### 2.1. Coagulation Factors Defects

The liver is the site of production of almost all coagulation factors (factors I, II, V, VII, IX, X, XI, and XII) except factors VIII which are produced in endothelial cells, and factor XIII which is produced in the bone marrow. With cirrhosis, there is a defect in the production of most factors that is reflected by a prolonged prothrombin time (PT) and activated partial thromboplastin time (aPTT) [17].

### 2.2. Thrombocytopenia

Patients with liver disease might have varying degrees of thrombocytopenia. In this population, three major mechanisms account for thrombocytopenia including impaired platelet production due to decrease hepatic synthesis of thrombopoietin, bone marrow suppression from infections and/or alcohol use, and increased platelet sequestration in the spleen [18].

### 2.3. Increased Fibrinolysis

Contributors to increased fibrinolysis in chronic liver disease patients are the increased level of tissue plasminogen activator (TPA) and the elevated levels of fibrin degradation products. This is possibly a result of the small number of hepatocytes and Kupffer cells which are thought to clear coagulation factors and fibrinolysis products from the circulation [19]. In addition, ascitic fluid which has a high fibrinolytic activity might drain via the thoracic duct to the systemic circulation contributing to the altered hemostasis [20, 21].

### 2.4. Prothrombotic Changes

The liver also synthesizes endogenous anticoagulation factors like protein C, protein S, and antithrombin III, as well as fibrinolytic factors which are not measured by routine blood assays. Moreover, the cirrhotic liver cannot clear von Willebrand Factor (vWF) which further adds to the prothrombotic effects of cirrhosis (reference).

## 3. Venous Thromboembolism (VTE) and Portal Vein Thrombosis (PVT) Prophylaxis in Patient with Liver Cirrhosis

Studies have demonstrated a 0.5-6.3% incidence of newly diagnosed DVT and PE among hospitalized patients with liver cirrhosis, a rate that is not different from that of patients without cirrhosis [22, 23]. Apart from venous thromboembolism, common thrombotic events affecting patients with liver disease are splanchnic vein thromboses including portal and hepatic vein thrombosis. Portal vein thrombosis (PVT) occurs in 8-25% of patient with decompensated liver cirrhosis as compared to only 1% of patients with compensated liver cirrhosis [23].

### 3.1. VTE Prophylaxis

The relationship between prophylactic anticoagulation and VTE in cirrhotics is controversial. While some studies showed lower incidence of VTE in patients treated with prophylactic anticoagulation [24], others failed to show any difference between nonrecipients and recipients of any form of prophylactic anticoagulation (mechanical or pharmacologic) [25]. However, prophylactic anticoagulation is appropriate for medical and surgical patients with liver cirrhosis after VTE risk factor assessment as studies showed no significant increase in the risk of bleeding in this patient category [24–26]. The VTE risk assessment in such patients can be performed using models such as the Padua prediction score [27], with exceptions to the use of prophylaxis being an overall low risk of VTE, severe thrombocytopenia, active bleeding, and high-risk varices. The treating physician's clinical judgment should take into consideration the above-mentioned data before starting VTE prophylaxis.

### 3.2. PVT Prophylaxis

PVT prophylaxis in patients with liver disease is not routinely used in clinical practice. A randomized clinical trial was done to assess the role of anticoagulation in the prevention of PVT. The RCT concluded that enoxaparin was safe and effective in preventing PVT in cirrhotic patients with a Child Turcotte Pugh (CTP) score of 7-10. PVT prophylaxis with enoxaparin appeared to delay the occurrence of hepatic decompensation and improve the transplant free survival [28].

## 4. Treatment Options for Thrombotic Diseases in Patients with Liver Cirrhosis

### 4.1. VTE

There is no consensus on the safety, monitoring, and efficacy of full dose anticoagulation to treat VTE in patients with liver cirrhosis. A general practice is to find a balance between the risks and benefits of anticoagulation. The risk of variceal bleeding is by far the most concerning potential complication in this patient population. In patient with increased risk of bleeding, treatment is controversial and anticoagulation should be avoided. Currently, in patients at low risk of bleeding, Warfarin is more often used than DOACs due to the lack of evidence on safety and efficacy of DOACs in this scenario. The choice and duration of therapy are usually based on a team-based decision between consultants [15, 26, 29].

### 4.2. Atrial Fibrillation Therapy

Data for anticoagulation in patients with atrial fibrillation and liver cirrhosis is also scanty. Ling Kuo et al. showed that the risk of ischemic stroke was lower in cirrhotic patients and atrial fibrillation treated with Warfarin, compared to patients receiving antiplatelet therapy or those untreated. On the other hand, the risk of intracranial hemorrhage (ICH) was similar between the three groups of patients. This reflects a net clinical benefit for Warfarin treatment in patients with cirrhosis and atrial fibrillation [30].

### 4.3. PVT Therapy

Patient with PVT in cirrhosis might experience spontaneous complete recanalization [31]. However, a number of studies have shown better rates of portal vein recanalization in patients treated with low molecular weight heparin or Warfarin compared to those who did not receive treatment, but without evidence of mortality benefit. Complete recanalization of the portal vein resulted in a trend towards less episodes of hepatic encephalopathy and lower rate of complications due to portal hypertension [32]. Senzolo et al. showed that, in patients with PVT treated with LMWH, lower rates of bleeding were reported with a ratio of one to five compared to patients that were left untreated [33]. Similarly, a recent meta-analysis from Italy compiled results of 8 RCTs with a total of 353 cirrhotic patients with PVT and showed a substantial benefit of anticoagulation with conventional agents. Patients with cirrhosis and PVT who received anticoagulation had significantly higher rates of recanalization and reduced clot progression, a significantly lower incidence of variceal bleeds, and a similar incidence of minor and major nonvariceal bleeding events compared to those who did not receive treatment [34]. Another two studies have suggested that LMWH is safe and effective in the treatment of PVT in patient with liver cirrhosis [35, 36]. Data about the role of DOACs in the treatment of cirrhotic PVT is limited. Several case reports indicated that Rivaroxaban and Apixaban may be used to treat PVT; however, their efficacy was reduced [37]. In a recent retrospective study, Nagaoki Y et al. evaluated PVT in fifty cirrhotic patients with variable CTP scores (CTP A: n = 29; CTP B: n = 16; CTP C: n = 5) that were treated with danaparoid sodium for two weeks and then randomized to receive either Warfarin or Edoxaban for a total of 6 months. Thirty patients received Warfarin with a target INR between 1.5 and 2, and 20 patients received Edoxaban that was dose adjusted to weight and creatinine clearance. The PVT volume was measured by dynamic computed tomography before, and periodically during treatment. After 6 months, the volume of PVT in patients treated with Edoxaban decreased from 1.42 cm^3^ to 0.42 cm^3^, signaling effective anticoagulation. Conversely, PVT volume was significantly higher after 6 months of treatment with Warfarin, increasing from 1.7 cm^3^ up to 2.85 cm^3^. As for the safety of anticoagulation, gastrointestinal bleeding occurred in 3 patients in the Edoxaban group compared to 2 patients in the Warfarin group. There was no significant difference among the two study arms regarding GI bleeding or any other adverse effects. Bleeding was successfully controlled in all patients using mechanical or pharmacological treatments. In addition, it is important to notice that none of the patients with CTP B developed GI bleed which can be an indicator that Edoxaban is possibly safe, specifically in this patient group. The same conclusion cannot be generalized to patients with CTP C, since none of the patients in this category received Edoxaban [38]. The results and the clinical utility of this study should be interpreted with caution, since the target INR was between 1.5 and 2, rather than the standard target between 2 and 3.

## 5. Classic Anticoagulants in Patients with Liver Cirrhosis

**Figure 1:**
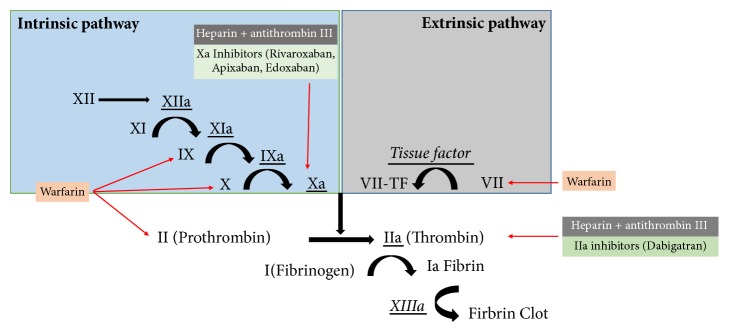
The effect on anticoagulants on the coagulation cascade.

In general, Heparin and LWMH function by potentiating the effect of antithrombin, which then inactivates several serial proteases, thus preventing the conversion of fibrinogen to fibrin (Figure 1).

Unfractionated heparin (UFH) potentiates the effect of antithrombin which inactivates thrombin (IIa) and other clotting factors (IX, X, XI, XII, and plasmin) and prevents the conversion of fibrinogen to fibrin.

LMWH also potentiates the effect of antithrombin to inactivate mainly factor Xa rather than thrombin (IIa) and subsequently prevent the conversion of fibrinogen to fibrin.

The effect of heparin in patients is monitored using the aPTT with a target prolongation of 1.5-2.5 times that of controls. Nonetheless, the decrease in antithrombin levels in patients with chronic liver disease may lead to falsely elevated aPTT, resulting in an undesired confusion over therapeutic levels in patients on heparin therapy. Alternatively, no routine monitoring is required for LMWH which is thought to carry a lower bleeding risk than unfractionated heparin in patients with cirrhosis. The latter is probably due to the higher sensitivity of cirrhotics to heparin [26]. When a prophylactic dose of enoxaparin was given for 48 weeks for prevention of PVT in patients with CTB class B and C, no increase in bleeding events was noticed compared to placebo [26]. Additionally, when therapeutic doses were given for long-term treatment of PVT, the risk of bleeding was not significantly increased [22, 23]. In comparison, therapeutic doses of heparin resulted in a significant drop in hemoglobin and platelets [39]. The advantage of anticoagulation with heparin derivatives is the availability of protamine sulfate as a reversal agent. Heparin and LWMH are to be withheld 4 and 12 hours respectively, prior to an invasive procedure.

Danaparoid sodium is an IV heparinoid agent that acts by inactivating factor Xa and thrombin. It is indicated for treatment of DVT and PE and can be used as a substitute for heparin in heparin induced thrombocytopenia. It has a comparatively lower bleeding risk and higher anticoagulant effect than heparin [40, 41]. In patients with liver cirrhosis, danaparoid sodium was found to be both, effective and relatively safe in treating PVT as it had no significant bleeding events when used for two weeks [42].

VKAs work by the inhibition of the synthesis of vitamin K dependent clotting factors II, VII, IX and X (Figure 1). Reversal agents include vitamin K, prothrombin complex concentrate (PCC), and fresh frozen plasma (FFP) in the setting were (PCC) is not available. Warfarin is stopped 5 days before any invasive procedure to prevent bleeding.

The baseline prolonged INR in liver cirrhosis leads to some controversy in initiating Warfarin on cirrhotic patients. According to current guidelines, the target therapeutic INR should be between 2 and 3 [43]. There are limited studies that assess the safety and efficacy of this approach in patients with cirrhosis. A recent study of 23 patients with liver cirrhosis showed that a target INR of 2-3 can be reached with Warfarin doses similar to those in noncirrhotic patients. The reduction of endogenous-thrombin-potential (ETP) was shown to reflect the effect of LMWH and Warfarin, and has been recently considered a potential monitoring parameter for anticoagulation in liver cirrhosis [44].

## 6. Direct Oral Anticoagulants (DOACs) in Patients with Liver Cirrhosis

**Figure 2:**
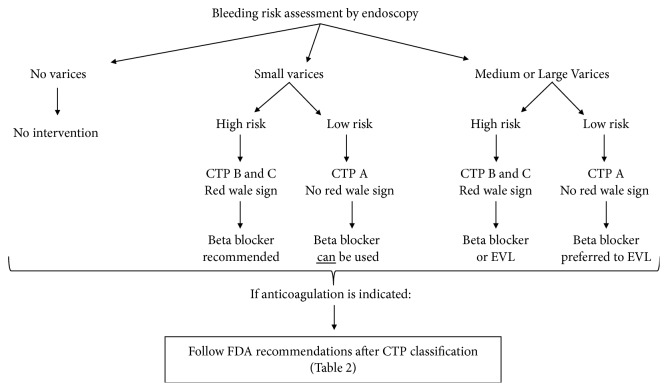
Recommended algorithm for DOACs therapy initiation in cirrhosis. CTP: Child Turcotte Pugh score. EVL: Endoscopic Variceal Ablation (see Table 2).

DOACs were developed to inhibit specific targets in the coagulation cascade. They are administered at fixed doses adjusted to kidney function, and routine monitoring for INR is not indicated. Indications for DOACs use include but are not limited to VTE prevention and treatment, stroke prevention in nonvalvular atrial fibrillation, and VTE prophylaxis after hip/knee surgeries. In the meantime, the approved DOACs in United States are three direct factor Xa inhibitors and one direct factor IIa inhibitor. DOACs represent a double challenge in patients with cirrhosis, first because of the impairment of the coagulation cascade, and second because of the possible effect of liver injury on drug metabolism. The hepatic elimination of Apixaban, Rivaroxaban, Edoxaban, and Dabigatran is 75%, 65%, 50%, and 20% respectively [45] (Table 1). Accordingly, the alteration of cytochrome P450 mediated metabolism, plasma protein binding, biliary secretion, and hepatorenal syndrome in patient with liver injury affect DOACs pharmacokinetics to various degrees. Child Pugh classification is recommended to assess the degree of hepatic injury and to determine the dosing of DOACs thereafter (Figure 2). On the other hand, the effect of DOACs on the liver in terms of liver injury adds to the complexity of the picture. Early clinical trials showed that Ximelagatran (factor IIa inhibitor) resulted in significant hepatotoxicity and subsequently the drug was withdrawn from the market. A following meta-analysis with more than 29 randomized controlled trials showed that the currently used DOACs have no significant risk of drug-induced liver injury [46].

Nonetheless, as experience with DOACs nurtures, there is accumulating evidence that the usage of DOACs is promising in cirrhotic patients in terms of efficacy and safety. Andexanet is a new medication that was recently US FDA approved in May 2018 for the reversal of anticoagulation by Rivaroxaban or Apixaban in life threatening and uncontrolled bleeding [47, 48]. New studies that are being conducted for DOACs antidotes will add more substratum to the use of DOACs in cirrhotic patients. Among the latest encouraging results, Idarucizumab to reverse the effect of Dabigatran and Ariprazine appears to reverse the effect of DOACs immediately after infusion [49, 50].

### 6.1. Dabigatran

1.5% to 3% of patients treated with Dabigatran have more than 3-fold rise in aminotransferase. This rate is similar to rates with Warfarin and lower than the rates with LMWH. Rare cases of jaundice and clinically apparent liver injury secondary to Dabigatran have been reported, but these cases were usually mild and self-limited [4, 51].

The approved prescribing guidance for Dabigatran does not include any specific recommendations for usage or dosing in patients with hepatic dysfunction [52]. An in vitro study compared the effect of Dabigatran on thrombin generation assays in healthy controls compared to cirrhotic patients' plasma. The addition of Dabigatran to cirrhotic patients' plasma resulted in a more pronounced anticoagulation effect compared to controls. In addition, higher reductions in thrombin generation time, reflecting a higher anticoagulation effect, seemed to be associated with cirrhosis severity, being highest in CTP C patients [53]. However, another in vivo study examining the effect of a single dose of Dabigatran 150 mg in healthy controls and patients with CTP B showed no difference in drug exposure or coagulation indices between the two groups [54].

### 6.2. Rivaroxaban

1.5% to 3% of patients treated with Rivaroxaban have more than 3-fold rise in aminotransferase, a rate that is similar to that of Warfarin and lower than that of LMWH. Rivaroxaban has been related to several instances of acute liver injury with jaundice, both hepatocellular and cholestatic or mixed patterns of liver injury have been reported. All reported cases of liver injury recovered after stopping Rivaroxaban [3, 55].

Rivaroxaban is not recommended for use in patients with CTP B or C cirrhosis or any liver disease associated with coagulopathy as per its approved guidance prescription [53, 56]. However, different studies have shown varying outcomes. A small study that evaluated the effect of Rivaroxaban in healthy controls compared to patients with varying degrees of hepatic disease excluding CTP C patients, revealed increased drug exposure in patients with CTB B cirrhosis relative to those with CTP A and healthy controls [57]. Alternatively, the in vitro study by Potze et al. demonstrated no difference in anticoagulation effect between healthy controls, CTP A or B cirrhosis. More so, Rivaroxaban resulted in a reduced anticoagulant effect in CTP C patients compared to controls, CTP A, and B patients [53]. One case reported that Rivaroxaban was safe and effective for the treatment of PVT in a patient with CTP A cirrhosis [58]. A case series examined 20 cirrhosis patients, nine of which received Rivaroxaban while eleven received Apixaban for PVT, VTE or stroke prophylaxis in atrial fibrillation. Outcomes were compared to a cohort study of traditional anticoagulation. The study showed no difference in the rates of major bleeding between the DOACs and the conventional anticoagulation arms [26]. To sum up, more clinical studies are needed to reach a conclusion about the efficacy and safety of DOACs in cirrhotic patients.

### 6.3. Apixaban

1% to 2% of patients treated with Apixaban have more than 3-fold rise in aminotransferase mainly in hepatocellular pattern; this rate is similar or lower than rates with Warfarin [59].

Apixaban requires no dose adjustment in patients with CTP A cirrhosis. While no specific recommendation is available for patients with CTP B cirrhosis, Apixaban is not recommended in patients with CTP C according to its approved prescribing guidance [60]. As mentioned previously, in the case series by Intagliata et al. 11 out 20 patients received Apixaban therapy for PVT, VTE, or stroke prophylaxis in atrial fibrillation. Similar rates of major bleeding events were observed between DOACs users and patients on conventional anticoagulation [26].

A recent small case series including CTP A patients showed that Apixaban may be safe and effective for selected patients with PVT [15].

### 6.4. Edoxaban

2% to 5% of patients treated with Edoxaban have more than 3-fold rise in aminotransferase. The rate is similar or lower than rates with Warfarin [2]. While no dose adjustment is necessary for Edoxaban use in patients with CTP A cirrhosis, the drug is not recommended in patients with CTP B and C [61]. A recent study examining the metabolism of one single dose of Edoxaban 15 mg in patients with CTP A and B found no difference in drug exposure between the 2 groups [62]. Large clinical trials including a more prolonged drug exposure are needed to further clarify the safety and efficacy of Edoxaban in patients with moderate to severe cirrhosis.

## 7. DOACs versus Traditional Anticoagulation in Patients with Liver Cirrhosis

To date, a limited number of clinical studies compared DOACs to traditional anticoagulation in patients with liver cirrhosis. One observational study on patients with cirrhosis compared 20 patients on DOACs to 19 patients on conventional anticoagulation therapy over a 3-year period. Among patients on DOACs therapy, 11 received Apixaban and 9 patients received Rivaroxaban. In comparison, 13 patients received VKA, and 6 patients received LMWH as traditional anticoagulation agents. The rate of any bleeding event was not significantly different between the two groups. Two major bleeding events occurred in the traditional anticoagulation group compared to one event in the DOACs group. The study concluded that DOACs have similar safety characteristics compared to traditional anticoagulation in patients with cirrhosis [26]. It is noteworthy to mention that in addition to its small sample size, this study only included CTP A and B patients and consequently, its results cannot be extrapolated to patients with CTP score C. The study's results are also in concordance with those of a meta-analysis suggesting no increased risk of drug-induced liver injury with DOACs use compared to conventional anticoagulation [46]. Another retrospective cohort study looked at cirrhotic patients with anticoagulation prescriptions for VTE treatment or stroke prophylaxis in atrial fibrillation. The study spanned over a 3-year period during which, 27 patients were prescribed DOACs (Rivaroxaban or Apixaban) and 18 participants were prescribed VKA and LMWH. Both groups had similar total bleeding events, however, there were significantly less major bleeding episodes in the DOACs group. Remarkably, compared to DOACs, traditional anticoagulation had a shorter time interval between initiation of therapy and a major bleeding event. While both groups had similar rates of gastrointestinal bleeding, three intracranial hemorrhage events occurred in the traditional anticoagulation arm while none took place in DOACs recipients. Otherwise, the rate of recurrent thrombosis was similar between both DOACs and traditional therapy groups, accounting to 1 event in each arm [29]. Despite the study's conclusion that DOACs were as effective as traditional anticoagulation agents with a better safety profile, there were major limitations worth noting such as the retrospective design of the study, its small sample size, and the limited number of CTP C patients (six) which restricted the applicability of those results in everyday practice.

## 8. Conclusion

DOACs are a breakthrough therapy for patients requiring anticoagulation. Among potential therapeutic advantages in cirrhotic patients, the benefits of DOACs include their quick onset of action, their simple dosage, and the fact that they do not need INR monitoring, thus sparing patients repeated laboratory testing and avoiding confusion in light of the existing altered coagulation parameters associated with cirrhosis. Still, the usage of DOACs in patients with cirrhosis must be evaluated on a case by case basis. To decrease the risk of possible bleed, control of underlying varices if present is to be attempted (Figure 2). To date, the indications to use DOACs in specific patient populations including cirrhotics are still an area of debate and further research. The majority of the available data is still considerably conflicting; however, results of trials in noncirrhotic patients are promising and suggest potential new indications for DOACs use. In summary, the evidence for safe use of DOACs in patients with hepatic impairment is still murky. Randomized controlled trials are needed to examine the efficacy, pharmacodynamics, and safety of DOACs especially in moderate to severe cirrhosis.

## Figures and Tables

**Table 1:** Route of elimination of DOACSs.

**DOAC**	**Liver elimination**	**Renal elimination**
Apixaban	75%	25%
Rivaroxaban	65%	35%
Edoxaban	50%	50%
Dabigatran	20%	80%

**Table 2:** FDA recommendation for DOACs usage in liver disease according to Child-Pugh class.

**Child-Pugh class A**	**Child-Pugh class B**	**Child-Pugh class C**
Dabigatran (no dose adjustment)	Use with caution (no dose adjustment)	Not recommended
Apixaban (no dose adjustment)	Use with caution (no dose adjustment)	Not recommended
Edoxaban (no dose adjustment)	Not recommended	Not recommended
Rivaroxaban (no dose adjustment)	Not recommended	Not recommended
